# Targeted deletion of *Insm2* in mice result in reduced insulin secretion and glucose intolerance

**DOI:** 10.1186/s12967-018-1665-6

**Published:** 2018-10-25

**Authors:** Lin Wang, Zhong Sheng Sun, Bingwu Xiang, Chi-ju Wei, Yan Wang, Kevin Sun, Guanjie Chen, Michael S. Lan, Gilberto N. Carmona, Abner L. Notkins, Tao Cai

**Affiliations:** 1 Institute of Genomic Medicine, Wenzhou Medical University, Wenzhou, China; 2 Beijing Institutes of Life Science, Chinese Academy of Sciences, Beijing, China; 3 Multidisciplinary Research Center, Shantou University, Shantou, Guangdong China; 4 Center for Research on Genomics and Global Health, NHGRI, NIH, Bethesda, MD USA; 5 Department of Pediatrics, Louisiana State University Health Sciences Center, New Orleans, LA USA; 6 Experimental Medicine Section, NIDCR, NIH, B30/Rm112, 30 Convent Dr., Bethesda, MD 20892 USA

**Keywords:** Human diabetes, Knockout mouse, Pancreatic islets, Glucose metabolism, Insulin secretion

## Abstract

**Background:**

Neurogenin3 (Ngn3) and neurogenic differentiation 1 (NeuroD1), two crucial transcriptional factors involved in human diabetes (OMIM: 601724) and islet development, have been previously found to directly target to the E-boxes of the insulinoma-associated 2 (*Insm2*) gene promoter, thereby activating the expression of *Insm2* in insulin-secretion cells. However, little is known about the function of *Insm2* in pancreatic islets and glucose metabolisms.

**Methods:**

Homozygous *Insm2*^−/−^ mice were generated by using the CRISPR-Cas9 method. Glucose-stimulated insulin secretion and islet morphology were analyzed by ELISA and immunostainings. Expression levels of *Insm2*-associated molecules were measured using quantitative RT-PCR and Western blots.

**Results:**

Fasting blood glucose levels of *Insm2*^−/−^ mice were higher than wild-type counterparts. *Insm2*^−/−^ mice also showed reduction in glucose tolerance and insulin/C-peptide levels when compared to the wild-type mice. RT-PCR and Western blot analysis revealed that expression of *Insm1* was significantly increased in *Insm2*^−/−^ mice, suggesting a compensatory response of the homolog gene *Insm1*. Similarly, transcriptional levels of *Ngn3* and *NeuroD1* were also increased in *Insm2*^−/−^ mice. Moreover, *Insm2*^−/−^ female mice showed a significantly decreased reproductive capacity.

**Conclusions:**

Our findings suggest that *Insm2* is important in glucose-stimulated insulin secretion and is involved in the development pathway of neuroendocrine tissues which are regulated by the transcription factors *Ngn3*, *NeuroD1* and *Insm1*.

## Background

Pancreatic endocrine cells play a key role in glucose metabolism by regulating the synthesis and secretions of islet hormones, such as insulin and glucagon. Mature islets contain five different types of endocrine cells. Ngn3, a proendocrine marker, is essential for islet cell development [1]. Biallelic mutations in *Ngn3* have been identified to cause permanent neonatal diabetes [2, 3]. NeuroD1, which is directly activated by Ngn3, participates in the maintenance and differentiation of mature islet cells [4]. Heterozygous mutations in *NeuroD1* have been identified to be associated with maturity-onset diabetes (OMIM: 601724) and type 2 diabetes mellitus [5, 6]. Although much efforts have been made to decipher the mechanism of differentiation and maintenance of pancreatic endocrine cells, the molecular basis of how Ngn3 and NeuroD1 function in the islets remains under investigation.

Previously we reported the isolation of two homologous genes, *INSM1* (a.k.a. IA-1) [7] and *INSM2* (INSM transcriptional repressor 2; a.k.a. IA-6) [8], from human pancreatic islet cells. Two additional genes, named PTPRN and PTPRN2 (a.k.a., IA-2 and IA-2beta), were also isolated from human islet cells, which have been turned out to be major autoantigens in patients with type 1 diabetes and are involved in the secretion of hormones and neurotransmitters [9, 10]. The encoded C_2_H_2_ zinc-finger proteins by *INSM1* and *INSM2* belong to the SNAIL/GFI1/INSM transcriptional repressor superfamily (i.e., SNAG-domain mediated transcription factors) that plays an important role in the developmental processes and molecular pathogenesis of various human conditions [11, 12]. For instance, mutations in *GFI1* and *GFI1B* have been identified in patients with neutropenia (OMIM: 600871) [13] and affected members of a family with platelet-type bleeding disorder-17 (OMIM: 604383) [14, 15], respectively.

Functional studies demonstrated both *INSM1* and *INSM2* are direct targets of Ngn3 and NeuroD1 [8, 16]. During the embryonic development, the *Insm1*^−/−^ knockout mice showed a significantly reduced insulin production, suggesting a key role for *Insm1* in islet β-cell differentiation and maturation [17, 18]. Recent studies demonstrated that Insm1 cooperates with Neurod1 and Foxa2 to maintain mature pancreatic beta-cell function [19]. *Insm2* also was found to be expressed in developing endocrine cells peaking from E11.5 to E13.5 and activated in Ngn3/NeuroD1-transduced pancreatic epithelial duct cells [8]. Numerous clinical studies showed that *Insm1* is a sensitive and highly specific marker for various tumors, such as neuroendocrine differentiation in primary lung neoplasms [20], Merkel cell carcinoma [21], small cell carcinoma of the prostate [22], head and neck tumors [23] as well as insulinoma.

To explore further the biological and pathophysiological role of *Insm2* in pancreatic islets, we developed an *Insm2*^−/−^ mouse using the CRSPR-Cas9 technique, and measured blood glucose, insulin and C-peptide levels of the *Insm2*^−/−^ mice [9]. Furthermore, we measured transcriptional levels of the transcription factors Ngn3, NeuroD1 as well as the homolog gene *Insm1* in Insm2^−/−^ mice.

## Methods

### Generation of *Insm2*-deficient mice

The pST1374-NLS-flag-linker-Cas9 (#44758) and pUC57-sgR (#51132) plasmids were commercially obtained (http://www.addgene.org/). Single guide RNA (sgRNA) was designed to target the coding region of the mouse *Insm2* gene (GenBank accession number: NM_020287.2). Mouse embryos were injected with Cas9 and sgRNA and then transferred into pseudo-pregnant mice to give birth to chimeric mice. Homozygous *Insm2*^−*/*−^ global knockout mice were obtained by breeding of *Insm2*^+*/*−^ mice and wild-type C57BL/6J. All experimental procedures were carried out in accordance with protocols approved by the NIDCR Animal Care and Use Committee (#12-641) and the Animal Usage Committee of Wenzhou Medical University (#31571301). Statistical analysis was performed using the Student’s *t* test for unpaired comparisons.

### Genotyping and quantitative real-time PCR

Primers for genotyping (forward: 5′-gtctcagctataaagcgggc-3′, reverse: 5′-aattggaacggatacaggga-3′) were located on flanking sides of the deleted region of *Insm2*. PCR products were genotyped by Sanger sequencing. Total RNAs were isolated from mouse brain and pancreas tissues using TRIZOL reagents (Life Technology, Rockville, MD, USA). The concentration of RNAs was determined by NANO 2000 (Thermal Scientific, Amarillo, TX, USA). Real-time PCR was performed using Mx3000p System with a two-step cycling program (Stratagene): 95 for 10 min, 40 cycles of 95 °C for 1 min and 60 °C for 30 s. Primers included for following genes: *Insm2* (forward: 5′-gctccggcagctcctacc-3′; reverse: 5′-ggctcctccggtgaggatt-3′), *Insm1* (5′-ggagtacgctgacccgttcg-3′; 5′-aagaccttggcgcactctgg-3′), *Ngn3* (5′-aagagcgagttggcatgagcaag-3′; 5′-gcgttggtccgctatgcgcag-3′), *NeuroD1* (5′-cttggccaagaactacatctgg-3′; 5′-ggagtagggatgcaccgggaa-3′), mouse Insulin 1 gene (5′-ccttagtgaccagctataatcagag-3′; 5′-cacttgtgggtcctccactt-3′) and Insulin 2 gene (5′-tcagcaagcaggaagcctatcttcc-3′; 5′- cacttgtgggtcctccactt-3′) [24]. *Gapdh* PCR product was used as a loading control.

### Western blots and antibodies

Proteins were isolated from mouse brain tissues for Western blots with following antibodies: anti-INSM2 rabbit antibody (1:800) [8], INSM1 rabbit antibody (1:1000, ab170876, Abcam, Shanghai, China), and anti-GAPDH antibody (1:1000, Santa Cruz (6C5), USA). The bands were visualized using enhanced chemiluminescence detection reagents (Applygen Technologies, Beijing, China) and detected by the FluorChem E imaging system (Cell Biosciences, Santa Clara, USA).

### Histological staining and immunostaining

Genotyped mice aged 10–23 weeks were sacrificed for isolation of pancreatic tissues, which were then treated in 10% formalin buffer for paraffin embedding. 5 μm thick sections on slides were stained with hematoxylin and eosin (H&E) for further analysis. Monoclonal anti-insulin antibody (1:250, Clone K36AC10, Sigma, USA) and anti-INSM2 rabbit antibody (1: 100) were also tested for immunostainings of mouse pancreas tissues [8].

### Intraperitoneal glucose tolerance test

Targeted mice aged 10–23 weeks were fasted for 14 h, followed by glucose injection (2 g/kg body weight via intraperitoneal injection). Venous blood was drawn from the tail vein at 0 (just before the injection), 15, 30, 60, 90 and 120 min after the injection of glucose. Blood glucose level was measured using a portable glucometer (Bayer, Elkhart, IN) as described previously [25].

### Insulin secretion

Glucose (3 g/kg body weight) was injected intraperitoneally into male and female mice aged 10–23 weeks. Insulin levels were measured using rat Insulin Ultrasensitive ELISA Kit and mouse C-peptide ELISA Kit (ALPCO, Salem, NH, USA). Blood samples were collected from the mouse orbital vein and centrifuged at the speed of 3000 rpm for 10 min at 4 °C and stored at − 80 °C until use.

## Results

### Characterization of *Insm2*^−/−^ mice

**Fig. 1:**
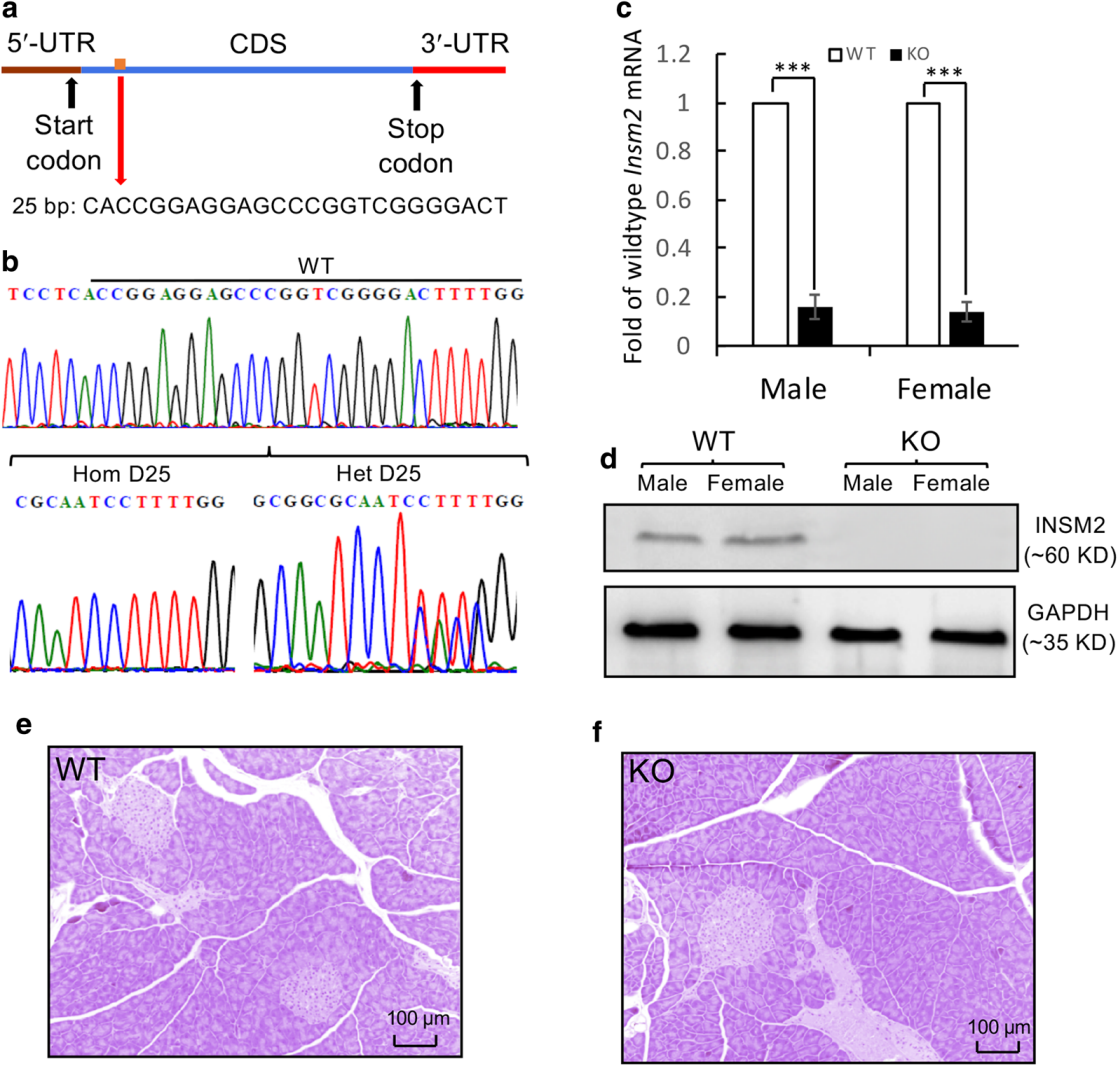
Genotyping and characterization of *Insm2*^−/−^ mice. **a** Targeted deletion of 25-bp (c.108_132delAGTCCCCGACCGGGCTCCTCCGGTG) in the coding sequence region (CDS) of *Insm2* (GenBank acc. no., NM_020287.2). **b** Sanger chromatograms show the target sequence regions in wild-type (WT), heterozygous (HET), and homozygous (HO) *Insm2*^−/−^ mice. **c** Quantitative RT-PCR analysis indicates that *Insm2* mRNA is markedly decreased in the *Insm2*^−/−^ mouse brain as compared to normal controls. **d** Western blotting shows no detectable INSM2 protein in *Insm2*^−/−^ mouse brain tissues. **e**, **f** H&E staining reveals no significant structure alterations in *Insm2*^−/−^ mouse pancreatic islets

The offsprings of the CRISP-Cas9 mice were genotyped by PCR and Sanger sequencing, which showed a 25-bp deletion in coding region of the sole exon of the *Insm2* gene (Fig. 1a). This deletion resulted in a frameshift mutation p.S36fsX67 (FWGPPASHLRRTTPSGARVAATAPAPAPRGRRAPSCAARSWSAACA LRSLRSPSPAPPP SAPRRPRP)*, thereby disrupting the reading frame of *Insm2*. Wild-type (*Insm2*^+/+^), heterozygous (*Insm2*^+*/*−^), and homozygous (*Insm2*^−/−^) mice were identified by tail DNA PCR and Sanger sequencing with the allele-specific primers (Fig. 1b).

Quantitative RT-PCR analysis of mRNAs extracted from the brain tissues of *Insm2*^−/−^ mice showed that the mRNA levels of *Insm2* were dramatically decreased, probably due to unstable *Insm2* mRNAs with the frameshifted deletion, compared to the wild type mice (Fig. 1c). The mRNA level of *Insm2* in pancreas tissue was also significantly decreased as compared to the wild type mice (not shown). Western blot analysis, using rabbit anti-INSM2 sera to measure protein expression level in mouse brain extracts, clearly showed no visible expression of INSM2 protein in the homozygous *Insm2*^−/−^ as compared to *Insm2*^+/+^ mice (Fig. 1d).

### Phenotype analysis

Physical examination of the *Insm2*^−/−^ mice revealed no gross development abnormalities. Body weight of *Insm2*^−/−^ male or female mice at 8, 16, and 24 weeks of age did not differ from the *Insm2*^+/+^ mice. However, fertility of *Insm2*^−/−^ female mice was significantly reduced. Specifically, after mating with *Insm2*^−/−^ male mice, of the 16 mating pairs of *Insm2*^−/−^ female mice, only five (31.25%) gave birth but with a smaller litter size (4 vs. 9 in normal mice, P < 0.0001). In a sharp contrast, all *Insm2*^−/−^ male mice had normal reproductive capacity when mating with *Insm2*^+/–^ female mice. However, the underlying pathogenesis of the significantly reduced fertility observed here needs further investigation.

**Fig. 2:**
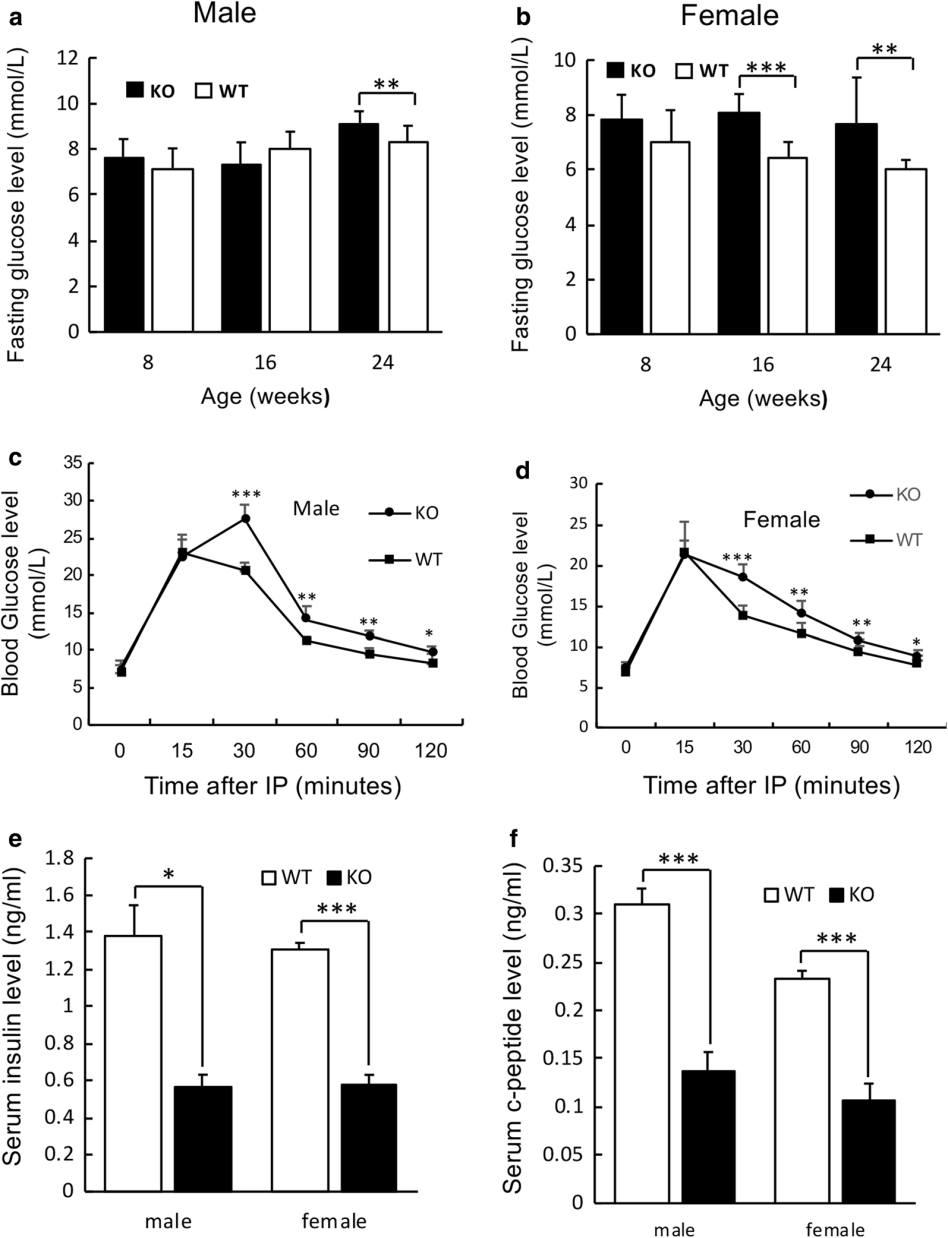
Analysis of glucose tolerance, insulin secretion, and *Insm2* homologous or regulatory genes. **a**, **b** Fasting glucose levels are elevated at 24 weeks *Insm2*^−/−^ male and at 16 and 24 weeks old *Insm2*^−/−^ female mice. Values are presented in mean ± SEM from eight animals per group in three separate experiments (**P < 0.01; ***P < 0.001). **c**, **d** Glucose tolerance tests in male and female *Insm2*^+/+^ and *Insm2*^−/−^ mice. After overnight fasting, d-glucose (2 g/kg body weight) was injected intraperitoneally, and blood glucose levels were measured at different times as indicated. Values are presented in mean ± SEM from 14 mice per group in three separate experiments (**P < 0.01; ***P < 0.001). **e**, **f** Blood insulin and C-peptide levels in response to intraperitoneal glucose in male and female *Insm2*^−/−^ mice aged 16–24 weeks were measured and compared to *Insm2*^+/+^ mice at 15 min. Blood samples were drawn from the tail vein using heparinized capillary tubes before and after glucose injection

Pancreatic tissue analysis using H&E staining in more than 30 slides did not show any significant alterations in terms of the number and size of islets in *Insm2*^−/−^ mice (Fig. 1e, f). However, fasting blood glucose levels of the *Insm2*^−/−^ male mice at 16 weeks of age (Fig. 2a) and female mice at 16- and 24 weeks of ages were elevated (Fig. 2b) as compared to the *Insm2*^+/+^ mice.

Glucose tolerance tests also showed significantly elevated glucose levels in both male and female *Insm2*^−/−^ mice (Fig. 2c, d). ELISA analysis showed that serum insulin levels in these mice were significantly lower than that in the *Insm2*^+/+^ mice (i.e., 0.56 ng/ml vs 1.37 ng/ml in male mice and 0.57 ng/ml vs 1.31 ng/ml in female mice, respectively, Fig. 2e). Similarly, basal blood C-peptide levels in the *Insm2*^−/−^ mice also were significantly decreased compared to the wild type mice (0.14 ng/ml vs 0.31 ng/ml in males and 0.11 ng/ml vs 0.23 ng/ml in female, respectively, Fig. 2f).

**Fig. 3:**
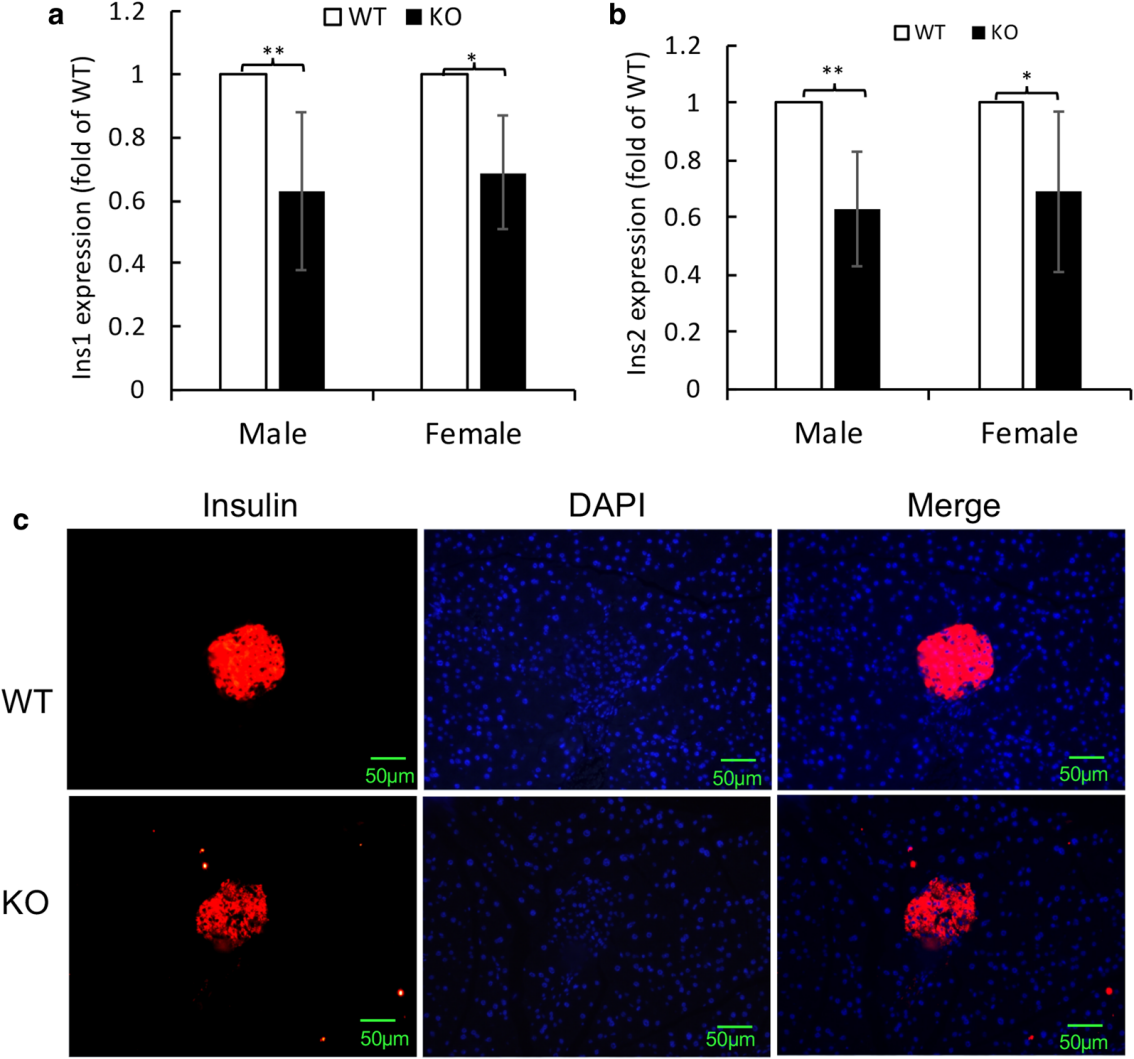
Insulin expression levels in pancreatic islet cells. **a**, **b** Expression of the *Ins1* and *Ins2* gene is decreased in the pancreas tissues of the *Insm2*^−/−^ mice compared to the wildtype controls. **c** Immunostainings show lower expression of insulin in the islet cells of *Insm2*^−/−^ mice compared to the wildtype controls

In fact, quantitative RT-PCR using primers of either the mouse insulin 1 gene (*Ins1*) or insulin 2 gene (*Ins2*) showed that their transcriptional levels in pancreatic tissues of *Insm2*^−/−^ mice were only 60–65% of the wild-type mice (Fig. 3a, b). Immunostainings using anti-mouse insulin antibody also showed reduced insulin expression in *Insm2*^−/−^ mouse islet beta cells compared to the wild-type islet cells (Fig. 3c).

### Expression levels of Insm2-associated regulators

**Fig. 4:**
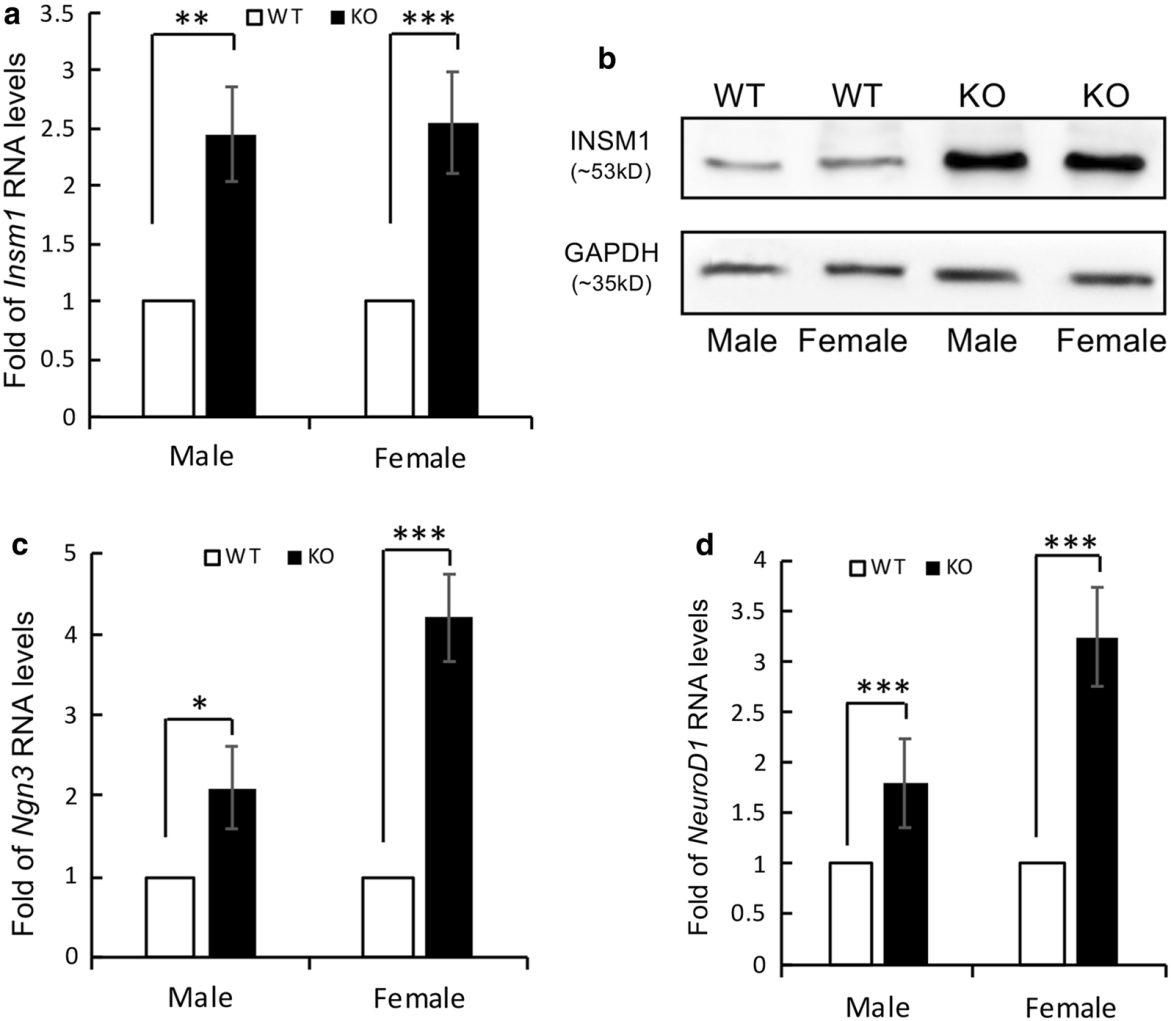
Expression levels of *Insm1*, *Ngn3*, and *NeuroD1*. **a**, **b** *Insm1* mRNA and its encoded protein levels extracted from brain tissues and measured by quantitative RT-PCR and Western blots. **c**, **d** Transcriptional levels of *Ngn3* and *NeuroD1* extracted from brains and analyzed by qRT-PCR. The results represent the average of three independent experiments in triplicates. Data are means ± SEM. *P < 0.01; **P < 0.001; ***P < 0.0001

Since *Insm2*-encoded protein shares significant homology as well as similar protein structure with *Insm1* [8], this suggested that the expression of *Insm1* might be increased as a compensatory response to the deficiency of *Insm2*. RT-PCR analysis of the *Insm2*^−/−^ brain tissue mRNAs showed a 2.5-fold increase of *Insm1* expression as compared to that of wild type mice (Fig. 4a). Western blotting with the anti-INSM1 antibody showed that the INSM1 protein levels in *Insm2*^−/−^ mice were 2 to 3-folds higher than that of wild type mice (Fig. 4b). Islet tissue was not examined because there is no detectable expression of Insm1/Ngn3/NeuroD1 in adult mouse islet cells.

Given that the transcription factors Ngn3 and NeuroD1 were previously found to promote *Insm2* expression via binding to the proximal E-boxes of the *Insm2* promoter [8], we examined their transcriptional response to the deletion of *Insm2*. RT-PCR analysis showed that expression levels of *Ngn3* and *NeuroD1* mRNAs were significantly increased in the *Insm2*^−/−^ mice compared to that of *Insm2*^+/+^ mice (Fig. 4c, d). Taken together, our findings suggest that the Ngn3 and NeuroD1 will elicit the expression of *Insm2* in a number of neuroendocrine tissues.

## Discussion

**Fig. 5:**
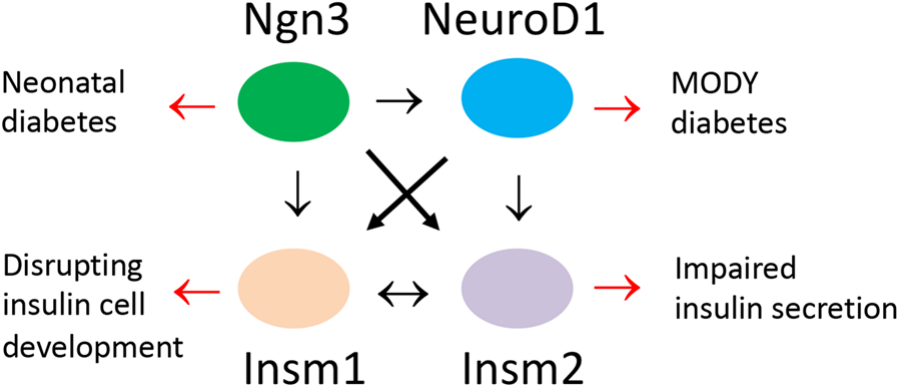
Interactions of several crucial transcriptional factors in pancreatic islet insulin-producing cells. Arrows in black indicate direct or regulatory interactions; arrows in red point to the phenotypic alterations due to deleterious mutations previously identified in affected individuals or specific knockout of the corresponding gene in mice

The human *INSM2* gene was mapped to chromosome 14q13.2 and showed a broader spatial-temporal expression pattern in neuroendocrine, heart, and liver tissues [8], whereas the *INSM1* expression was restricted in neuroendocrine tissues and tumors [26, 27]. INSM2 belongs to the SNAI1 (OMIM: 604238)/GFI1 (OMIM: 600871)/INSM1 (OMIM: 600010) family of transcriptional repressors (OMIM: 614027). Intriguingly, the mouse *Insm2* gene was found to be methylated and silenced in liver tumors of SV40 T antigen transgenic mice [28]. In contrast to the earlier death of *Insm1*^−/−^ mice with disrupted development of insulin-producing beta cells [17], the phenotypes we observed in *Insm2*^−/−^ mice were relatively subtle. Although no obvious diabetic symptoms were observed in *Insm2*^−/−^ mice, elevated fasting glucose, elevated glucose levels in glucose tolerance test, lower serum insulin secretion, and lower C-peptide levels are indicators that deletion of *Insm2* affects pancreatic islet cell functions (Fig. 5). Understanding of the regulatory mechanisms of critical transcriptional factors, such as Ngn3, NeuroD1, and Insm1, in islet development is important for making insulin-producing cells for prospective stem cell therapy to treat diabetes [29–31].

In depth analyses of *Insm1*, *Ngn3*, and *NeuroD1* in *Insm2*^−/−^ mice revealed that their increased expressions could be a compensatory response to the deficiency of *Insm2* because both *Insm1* and *Insm2* share a common activation pathway through Ngn3 and NeuroD1 in islet cells [8]. Previous study also demonstrated that endogenous *Insm2* expression was activated in Ngn3/NeuroD1-transduced pancreatic epithelial duct cells [8]. Therefore, the phenotype severity in pancreatic islets of *Insm2*^−/−^ mice could be lessened through the increased *Insm1* expression. In fact, similar to the expression pattern of *Insm2* in brain [8], *Ngn3* and *NeuroD1* are also abundantly expressed in brain tissues, especially in developing and adult hippocampus [32, 33]. Therefore, the increased expression levels of *Ngn3* and *NeuroD1* in brain tissues should be a compensatory response to the deletion of *Insm2* in mice. As previously reported, Ngn3 and NeuroD1 positively regulate *Insm2* expression through the binding of the E-box of the *Insm2* promoter [8]. It would be interesting to search additional insulin-pathway related phenotypes in the double knockout mice harboring homozygous *Insm2*^−/−^ and heterozygous *Insm1*^+/−^ alleles.

Furthermore, genetic analysis by GWAS in a cohort of Africa Americans with type 2 diabetes revealed a significant association of the disease to a SNP (rs1952392, MAF = 0.0188; P < 0.001) at the proximal promoter region of the human *INSM2* gene (personal communication) [34]. A previous genetic study also showed an association of microsatellite polymorphisms at 14q13.2 with type 2 diabetes mellitus in Latvian and Finnish populations [35]. Interestingly, INSM2 was recently found to upregulate the expression of the ultraconserved (uc) RNA uc.372, which in turn suppresses the maturation of *miR*-*195*/*miR*-*4668* to regulate expression of genes related to lipid synthesis and uptake in liver [19]. Given the risk of non-alcoholic fatty liver disease in type 2 diabetes mellitus (see recent meta-analysis) [36, 37], it is worth noting whether the deletion of *Insm2* in mice affects the hepatic lipid accumulation and thus attributes to the risk of type 2 diabetes.

Based on the important role of the *INSM2* gene in neuroendocrine tissues as well as the preliminary clinical findings, potential mutations and altered expressions of the *INSM2* gene in neuroendocrine-associated disorders, such as diabetes and infertility, can be screened utilizing next-generation sequencing technologies. In a cancer-related study, *Insm2* was found to be methylated and silenced in liver tumors of SV40 T antigen transgenic mice [28], suggesting its role in tumor inhibition. Among 64 different mammalian cell lines, intriguingly, INSM2 appears to be exclusively expressed in SH-SY5Y cells (a neuroblastoma cell line, Human Protein Atlas, https://www.proteinatlas.org). Further studies are needed to learn whether or not INSM2 plays a role in tumorigenesis and functions as a prognostic marker in neuroblastoma.

## Conclusion

In conclusion, we demonstrated that deletion of *Insm2* affects glucose-stimulated insulin secretion and glucose tolerance. We also showed that *Insm2* is involved in the Ngn3/NeuroD1 pathway in neuroendocrine tissues. Whether *Insm2*^−/−^ mice induce islet cell differentiation and proliferation defects and/or develop type 2 diabetes, further experiments under more challenging conditions, such as high-fat and high-sugar diet [38, 39], are required to test this hypothesis.
